# Associations Between the Five Factor Model of Personality and Dementia-Related Anxiety

**DOI:** 10.1093/geroni/igaa057.1242

**Published:** 2020-12-16

**Authors:** Stacy Yun, Lisa Stone, Evan Furr, Molly Maxfield

**Affiliations:** 1 University of Colorado Colorado Springs, Colorado Springs, Colorado, United States; 2 Arizona State University, Phoenix, Arizona, United States

## Abstract

The Five Factor Model (FFM) of normative personality is predictive of long-term outcomes, including well-being and anxiety. For example, people with anxiety disorders often report high Neuroticism and low Conscientiousness (Kotov et al., 2010). Dementia-related anxiety (DRA) is concern about developing dementia that can occur in individuals of any age and cognitive status (Kessler et al., 2012). This study assessed associations between the FFM and DRA and the extent to which other factors, such as demographics and variables related to DRA (i.e., external locus of control and lacking knowledge of dementia), contributed to relationships. Participants (N = 664; aged 18 to 81; M = 30.24) completed measures of the FFM, DRA, locus of control, and dementia knowledge. Hierarchical regression (block 1: basic demographics, block 2: DRA-related variables, and block 3: FFM) was computed. The set of predictors explained 17.9% of the variance in DRA, F(14, 623) = 9.69, p < 001. Being older, partnered, low on Conscientiousness and Openness, and having greater external locus of control and less dementia knowledge predicted higher DRA (p-values < .05). Surprisingly, Neuroticism was not predictive of DRA after controlling for demographic and DRA-related factors, indicating that the trait-like tendency towards emotional instability does not explain DRA. Longitudinal research can explore the course of relationships among Conscientiousness, Openness, and DRA over time to further examine significant effects of age, as expressions of personality change across the lifespan. Research targeting potentially modifiable factors (i.e., dementia knowledge) could help identify methods of reducing DRA.

